# Baicalin prevents the up‐regulation of TRPV1 in dorsal root ganglion and attenuates chronic neuropathic pain

**DOI:** 10.1002/vms3.318

**Published:** 2020-07-01

**Authors:** Zheyin Wang, Diyang Ling, Chenxiang Wu, Jian Han, Yan Zhao

**Affiliations:** ^1^ Department of Pain Medicine Shenzhen People's Hospital The First Affiliated Hospital of Southern University of Science and Technology Shenzhen China

**Keywords:** baicalin, Chinese medicine, DRG, neuropathic pain, TRPA1, TRPV1

## Abstract

**Background:**

Neuropathic pain is a major public health problem because it has a considerable impact on life quality of patients. TRP channels from dorsal root ganglion (DRG) play a crucial role in facilitating pain transmission at peripheral and spinal sites. Baicalin has neuroprotective effects and improves the pathological and behavioural outcomes of various types of nerve injury. The present study aims to examine the analgesic effects of baicalin on chronic neuropathic pain.

**Methods:**

Neuropathic pain animal model was created by chronic constriction injury of the sciatic nerve (CCI). Behavioural tests were performed by von Frey and hot plate tests. mRNA and protein expression levels were examined by quantitative RT‐PCR and western blot.

**Results:**

Consecutive intraperitoneal administration of baicalin for 16 days reduced the mechanical and thermal nociceptive responses induced by CCI surgery in a dose‐dependent manner. The mRNA expression levels of *Trpv1* and *Trpa1* were significantly increased in the DRG of CCI rats. Moreover baicalin administration reversed TRPV1 expression and phosphorylation of ERK in DRG neurons after peripheral nerve injury.

**Conclusions:**

Our results suggested that baicalin may ameliorate neuropathic pain by suppressing TRPV1 up‐regulation and ERK phosphorylation in DRG of neuropathic pain rats. Baicalin has a significant analgesic effect on alleviating neuropathic pain and thus may serve as a therapeutic approach for neuropathic pain.

## Graphical abstract

Consecutive intraperitoneal administration of baicalin for 16 days reduced the mechanical and thermal nociceptive responses induced by CCI surgery in a dose‐dependent manner. Baicalin administration reversed TRPV1 expression and phosphorylation of ERK in DRG neurons after peripheral nerve injury. Baicalin ameliorates neuropathic pain by suppressing TRPV1 up‐regulation and ERK phosphorylation in DRG of neuropathic pain rats.

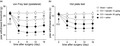

## 1 INTRODUCTION

Neuropathic pain is a major public health problem because it has a considerable impact on life quality of patients. Neuropathic pain caused by a lesion or disease of the somatosensory nervous system, which causes unpleasant and abnormal sensation (dysesthesia), an increased response to painful stimuli (hyperalgesia), and pain in response to a stimulus that does not normally provoke pain (allodynia) (Finnerup et al., 2016). Therefore, it is warranted to develop novel effective analgesics for neuropathic pain relief and treatment.

Most of the transient receptor potential (TRP) ion channels are calcium‐permeable, non‐selective cation channels (Montell & Rubin, 1989). TRP channels are characterized by various activation mechanisms (Nilius, 2007). It has been reported that some of the TRP channel superfamily ion channels are involved in nociception and thermosensation, both in the peripheral and central nervous systems (Caterina et al., 1997). TRP vanilloid 1 (TRPV1) is activated by multiple mechanisms, such as noxious heat (>43°C), protons and some pungent chemicals such as capsaicin (Caterina et al., 1997). TRP ankyrin 1 (TRPA1) is widely expressed in sensory neurons which are involved in pain pathway. TRPA1 functions as a sensor of environmental and endogenous chemical irritants, such as 4‐hydroxynonena, allyl isothiocyanate (AITC) and icillin (Cho, Jeong, Choi, Lee, & Jang, 2012). TRPV1 and TRPA1 are shown to have a crucial role in nociceptive transmission under pathological forms of pain (Chen, Willcockson, and Valtschanoff 2009). Moreover, it is clear that spinal synaptic plasticity is an important procedure for the pain transition from acute to chronic in which TRPV1 and TRPA1 play critical roles presynaptically and postsynaptically (Choi, Lim, Yoo, Kim, & Hwang, 2016). TRPV1 and TRPA1 expression in nociceptors are altered in different models of neuropathy under pathological conditions (Biggs et al., 2007; Kim et al., 2008).

Plant natural products are well‐known in the therapy of chronic comorbidities (e.g. diabetes, cancer and pain) as traditional Chinese medicine (Yuan, Guo, Liu, Sun, & Zhang, 2015). Baicalin (7‐glucuronic acid, 5, 6‐dihydroxyflavone), a flavonoid compound extracted from Huang Qin, possesses antioxidant properties (Chou, Chang, Li, Wong, & Yang, 2003) and has an analgesic effect on nitroglycerin‐induced migraine in rats (Sun et al., 2017) and neuropathic pain in spinal nerve ligation rats (Cherng et al., 2014). These studies suggested that the potential effects of baicalin in the relief of pain originated from peripheral nervous system.

However, the putative impact of baicalin on neuropathic pain and the underlying mechanisms remain unclear. Therefore, we hypothesized that baicalin may ameliorate neuropathic pain via TRP channels. Here, detailed experiments were carried out to investigate the analgesic role of baicalin on neuropathic pain. Our results demonstrated that baicalin administration ameliorates both neuropathic pain and TRPV1 expression in DRG neurons after peripheral nerve injury in rats. Our data highlighted the potential therapeutic role of baicalin in the treatment of neuropathic pain.

## 2 METHODS AND MATERIALS

### 2.1 Animals

Male Sprague–Dawley rats weighing 200–220 g were used for all experiments. The animals were housed by 2–3 rats/cage under a standard 12 hr light/dark cycle at 23 ± 1°C, with free access to water and chow. Before experiments, all animals were adapted to the experimental circumstances for 5–7 days. All experimental procedures were conducted in strict adherence to the Guide for the Care and Use of Laboratory Animals and approved by the Animal Care and Use Committee of XXX. All the surgeries were performed with the rats under deep anaesthesia following intraperitoneal administration of sodium pentobarbital (50 mg/kg). The chronic neuropathic pain model was established based on chronic constriction injury of the sciatic nerve (CCI) as previous reported (Sun et al., 2018). Briefly, rats were anesthetized with 4% chloral hydrate (10 ml/kg, i.p.), the right sciatic nerve was exposed at the mid‐thigh level and approached to the sciatic trifurcation. Four constrictive ligatures (4‐0 surgical suture) were tied around the nerve at the distal end close to the bifid site at intervals of about 1.0 mm. The ligature was considered complete when a local mild muscular contraction of the leg could be clearly seen. All operations were finished by the same operator. In the sham group, the sciatic nerve was exposed but not tied. The mechanical withdrawal threshold and the thermal withdrawal latency of every rat was measured as an assessment of nociception prior to the CCI operation and at days 4, 8, 12 and 16 after the CCI operation.

### 2.2 Drug administration and experimental design

Baicalin was purchased from Wako (Wako Pure Chemical Industries, Osaka, Japan) and dissolved in 2% DMSO and saline. The stock solution was filtered through a 0.22 μm membrane filter before use. For behavioural experiments, rats were randomly divided into four groups (10 rats in each group): a sham group, a CCI group, a CCI group treated with baicalin (15 μg/Kg) and a CCI group treated with baicalin (30 μg/Kg). Rat received injection (i.p.) of baicalin daily at 24 hr intervals, while sham and CCI animals received the same volume of DMSO in saline.

### 2.3 Nociceptive tests

#### 2.3.1 Mechanical allodynia

All the behavioural tests were performed between 9:00 a.m. and 6:00 p.m. Animals were habituated in the testing apparatus for at least 30 min until they calmed down before behavioural testing. Von Frey filaments were applied with increased forces from 0.4 to 60.0 g until the paw withdrawal threshold was detected. Briefly, the von Frey hairs were pressed vertically on the hind plantar surface for approximately 4–5 s; each filament was used ten times; and a 5‐min interval was left between the different forces. The minimal force that caused lifting or licking responses at least five times was considered as the paw withdrawal threshold (Hao et al., 2019; Sun et al., 2019).

### 2.4 Thermal hyperalgesia

Thermal hyperalgesia was assessed using a hot plate analgesia meter (BIO‐CHP, Bioseb, France). Rat was placed on a hot plate which is maintained at a temperature of 53 ± 1°C. Response latency either lifting or licking was recorded by means of an electronic timer. To prevent tissue damage, a cut‐off time of 30 s was adopted. Each rat was measured at least three times with a 10‐min interval and the average value was calculated. Lifting for normal locomotion was excluded. Behavioural testing was carried out with the experimenters blinded to treatment conditions (Hao et al., 2019; Sun et al., 2019).

### 2.5 Quantitative real‐time RT‐PCR

Rats from each group before and after 4, 8, 12 and 16 days CCI operation were anaesthetized (i.p.) and sacrificed. The ipsilateral lumbar (L) 4, 5, 6 DRGs were collected from the three levels. DRGs were pooled and placed in tubes with Trizol reagent (Sigma‐Aldrich, USA). RNA isolation was followed by chloroform extraction and isopropanol precipitation. cDNA was synthesized using the iScript™ cDNA Synthesis kit (Biorad, USA). Copy numbers of rat genes were determined by quantitative RT‐PCR (qRT‐PCR) using SYBR Green MASTER (Invitrogen, USA) following the manufacturer's protocol. The results were standardized for comparison by measuring levels of *Gapdh* mRNA in each sample. The primers were used according to the previous report (Pohoczky et al., 2016; Xue, Jong, Chen, & Schumacher, 2007).

### 2.6 Protein preparation and western immune blotting

Each sample was homogenized and centrifuged in lysis buffer (150 mM NaCl, 50 mM Tris‐HCl, pH 7.4, 1% Triton‐100, 1% sodium deoxycholate, 0.1% SDS) to extract the protein. The extracted protein in lysis buffer was then kept at −80°C. Equal amounts of protein (20 µg) were separated by 10% Tris‐Tricine SDS‐PAGE and transferred onto polyvinilidene difluoride (PVDF) membranes. PVDF membranes were then blocked in 10% non‐fat milk for 1 hr at room temperature followed by overnight incubation at 4ºC with rabbit anti‐TRPV1 (1:1,000; Abcam, Massachusetts, USA), ERK (1:200; Abcam, Massachusetts, USA), pERK (1:500; Abcam, Massachusetts, USA) and GAPDH (1:5,000; Abcam, Massachusetts, USA) primary antibody. PVDF membranes were then incubated for 30 min at room temperature with goat anti‐rabbit polyclonal IgG (1:2000, Abcam, Massachusetts, USA). Among these steps, PVDF membrane were rinsed with TBS‐T 3 times for 10 min. Protein bands were visualized with the ECL kit (Amersham Pharmacia Biotech, Piscataway, USA) and then detected by using the ChemiDoc Imaging System (Bio‐Rad, Berkeley, USA). The density of the bands was quantified after background subtraction and then normalized with respect to the GAPDH or ERK levels.

### 2.7 Statistical analysis

Group data are presented as mean ± standard error mean (*SEM*). Statistical analysis was carried out with repeated measures ANOVA followed by LSD post hoc test or one‐way ANOVA followed by two‐tailed *t*‐test with Bonferroni correction using Origin 10.0 software. *p* values less than .05 or .01 were considered having a significant difference.

## 3 RESULTS

### 3.1 Establishment of the neuropathic pain rat model

**FIGURE 1:**
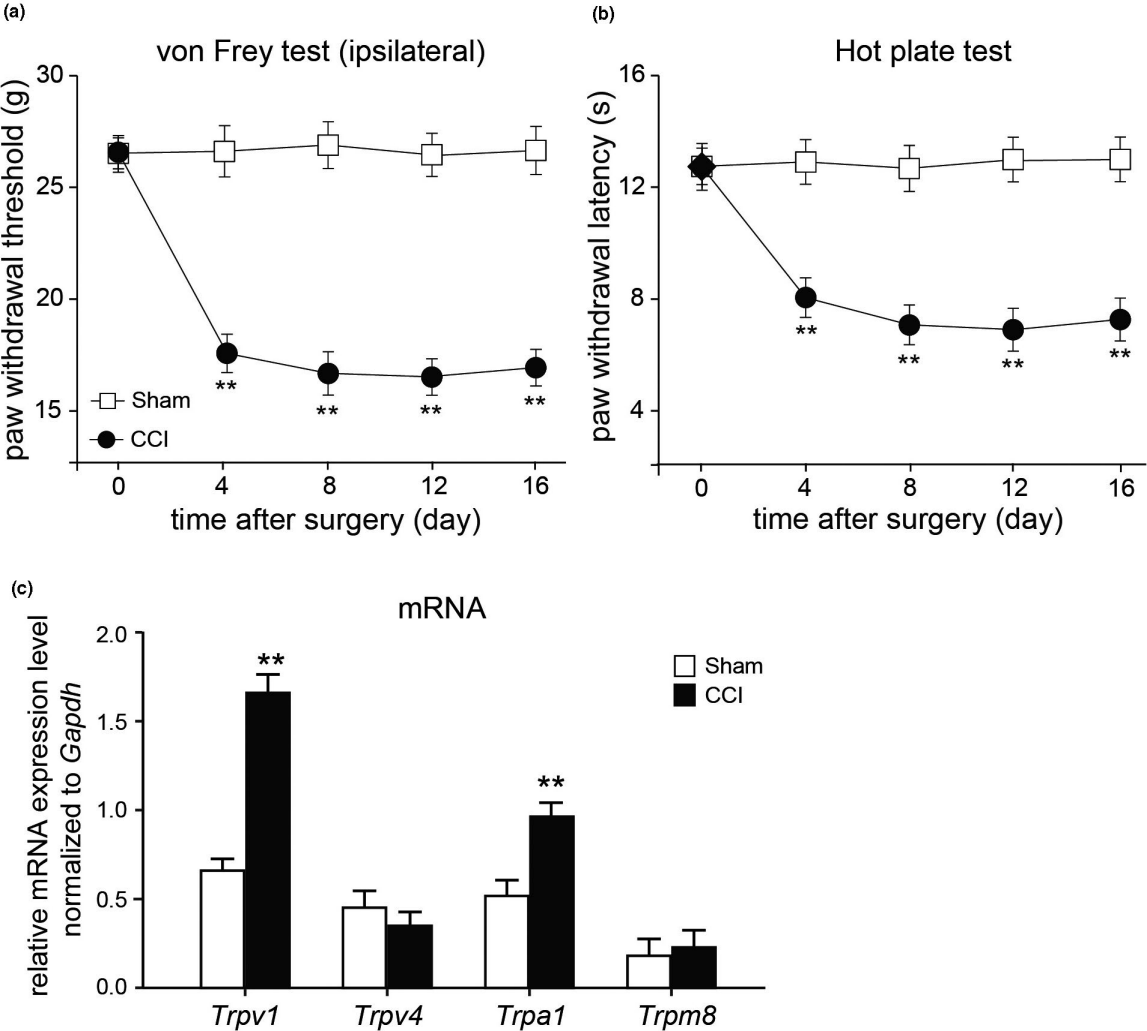
Mechanical tests in rats with or without CCI surgery. (a and b) Mechanical (a) and thermal (b) tests in rats after sham or chronic constriction injury of the sciatic nerve (CCI). Mean ± *SEM*, *n* = 8; **p < .01 versus sham group. Repeated measures ANOVA followed by LSD post hoc test. (c) The mRNA expression of *Trpv1*, *Trpv4*, *Trpa1* and *Trpm8* in DRG from rats received sham or CCI surgery for 16 days. Mean ± *SEM*, *n* = 8; **p < .01 versus sham group. Unpaired Student's *t*‐test

First of all, chronic constriction injury of the sciatic nerve was performed to create neuropathic pain model in rats. After the onset of CCI model rats, we tested the mechanical thresholds and paw withdrawal latencies in response to thermal stimulation in rats from sham and CCI groups. The CCI model rats began to show obvious mechanical sensitivity (ipsilateral) 1 day after CCI surgery (Figure 1a, *p* < .01, *n* = 8, repeated ANOVA). In parallel, a dramatic thermal hyperalgesia to noxious plantar heat stimulation was observed in CCI model rats (Figure 1b, *p* < .01, *n* = 8, repeated ANOVA). These results suggested that the neuropathic pain model was successfully established in rat after CCI surgery. It is well‐documented that several TRP channels are involved in neuropathic pain. We next examined the mRNA expression of *Trpv1*, *Trpv4*, *Trpa1* and *Trpm8* in DRGs of rat 16 days after CCI surgery. Our results indicated that the mRNA levels of *Trpv1* and *Trpa1* in DRG were significantly increased in rats after peripheral nerve injury (Figure 1c, *p* < .01, *n* = 8, Unpaired Student's *t*‐test).

### 3.2 Baicalin reversed the progress of neuropathic pain in CCI rats

**FIGURE 2:**
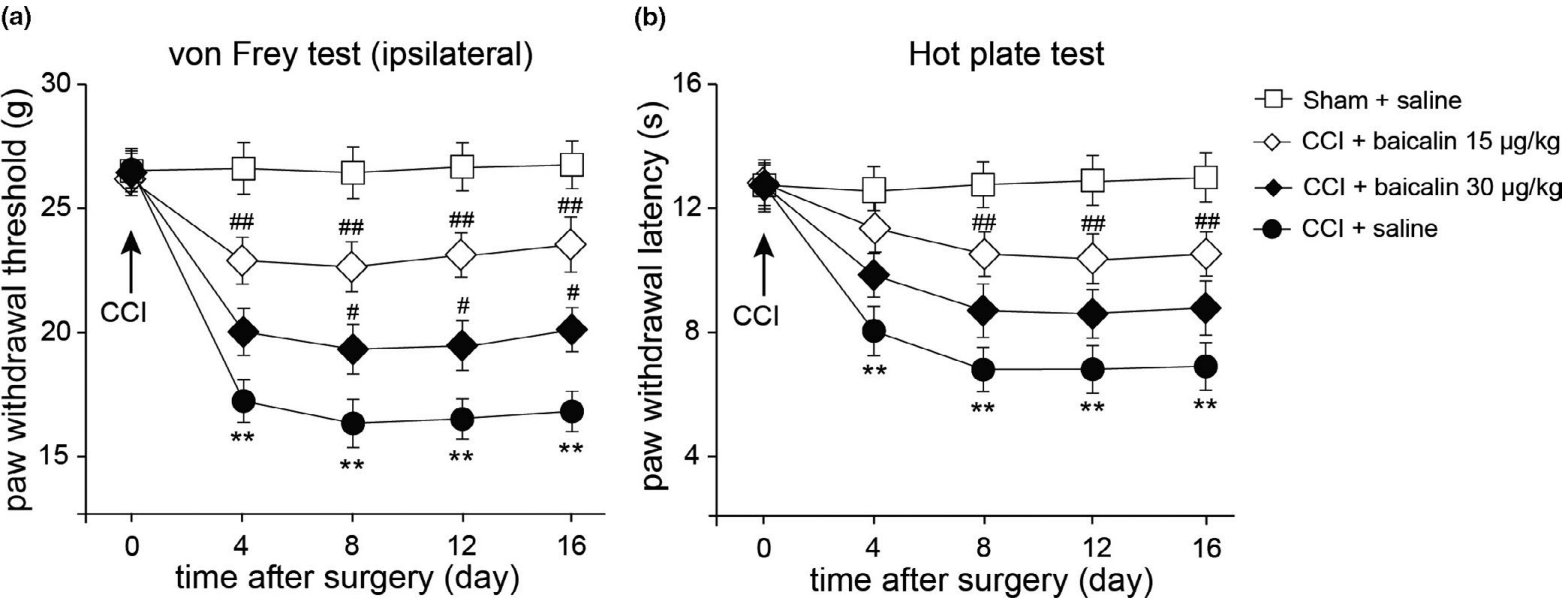
Reduction of mechanical allodynia and thermal hyperalgesia by consecutive injection of baicalin in rats after peripheral nerve injury. (a and b) Prevention of mechanical allodynia (a) and thermal hyperalgesia (b) by repeated injection of Baicalin. Baicalin (15 or 30 μg/kg) was repeatedly injected (i.p., every day). Paw withdrawal threshold in the von Frey tests and paw licking latency in the hot plate tests were measured in rats before and after surgery. Mean ± *SEM*, *n* = 8, **p < .01 versus sham group; ^#^ *p* < .05, ^##^ *p* < .01 versus CCI group. Repeated measures ANOVA followed by LSD post hoc test

We next explored the analgesic effects of baicalin administration intraperitoneally (i.p.) on CCI rats. Baicalin (15 or 30 μg/kg) was injected (i.p.) every day in CCI rats. Our data showed that the mechanical thresholds (ipsilateral) in rats after CCI surgery were decreased less in either CCI + baicalin 15 μg/kg or CCI + baicalin 30 μg/kg group, compared to CCI group (Figure 2a, *p* < .05 or *p* < .01, *n* = 8, repeated ANOVA). Moreover the paw withdrawal latency in response to thermal stimulation were significantly elevated in either CCI + baicalin 15 μg/kg or CCI + baicalin 30 μg/kg group, compared to CCI + saline group (Figure 2b, *p* < .05 or *p* < .01, *n* = 8, repeated ANOVA). These results suggested that baicalin may prevent the progress of neuropathic pain in rats upon peripheral nerve injury.

### 3.3 Baicalin suppressed the up‐regulation of *Trpv1* mRNA expression in DRG of CCI rats

**FIGURE 3:**
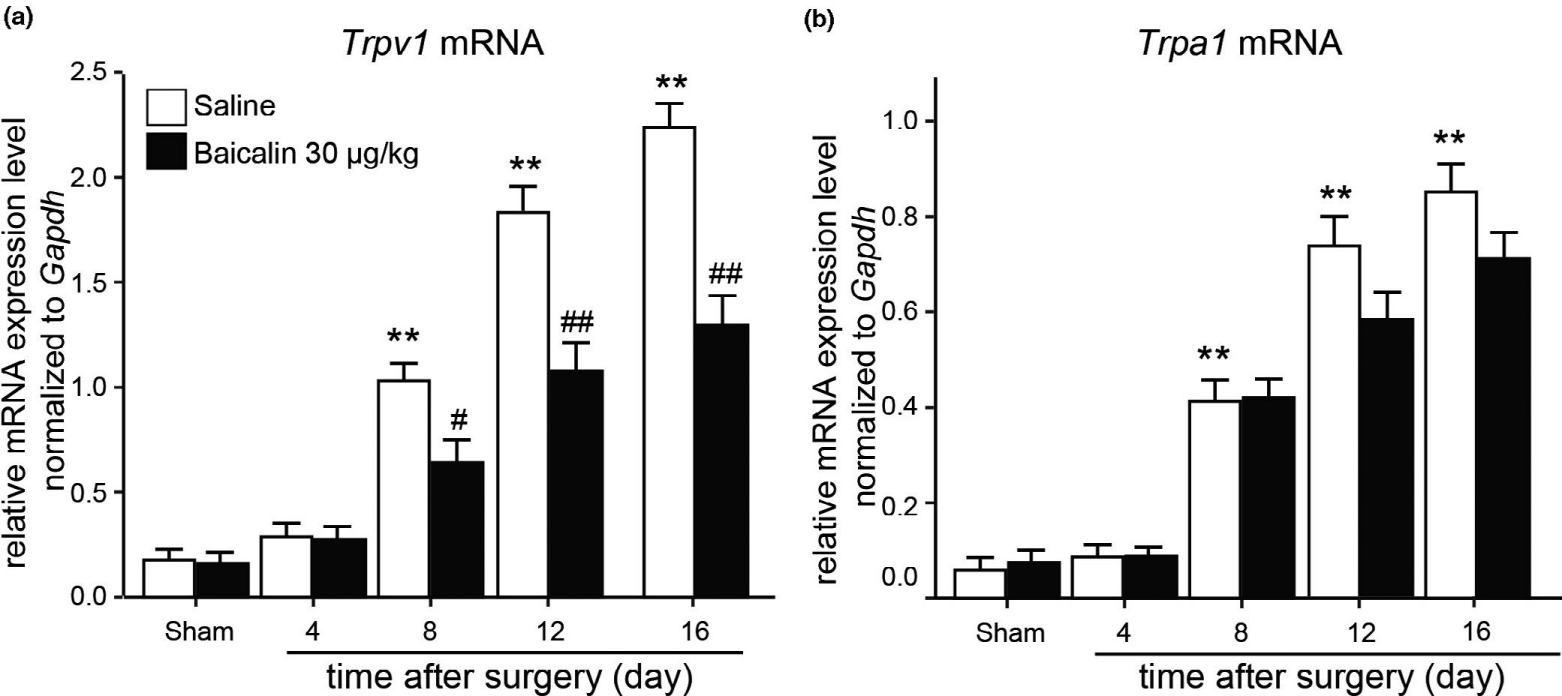
mRNA expression levels of *Trpv1* and *Trpa1* in DRG of CCI rats after baicalin treatment. (a and b) *Trpv1* (A) and *Trpa1* (B) mRNA expression levels in DRG of CCI rats before and after saline or baicalin treatment for 4, 8, 12 and 16 days. Mean ± *SEM*, *n* = 8; **p < .01 versus sham group. ^#^ *p* < .05, ^##^ *p* < .01 versus saline group. One‐way ANOVA followed by two‐tailed *t*‐test with Bonferroni correction

To explore the potential mechanisms which baicalin involved in the prevention of neuropathic pain, we examined *Trpv1* and *Trpa1* mRNA expression in DRG of rats with peripheral nerve injury using quantitative RT‐PCR method. Our results demonstrated that *Trpv1* and *Trpa1* mRNA expression levels were dramatically increased in DRG of CCI rats 8, 12 and 16 days after surgery, but only *Trpv1* mRNA expression was significantly suppressed by repeated baicalin administration (Figure 3b, *p* < .05 or *p* < .01, *n* = 8, one‐way ANOVA) in DRG of CCI rats 8, 12 and 16 days after surgery. These results revealed that baicalin suppresses *Trpv1* mRNA expression up‐regulation in rat DRG after peripheral nerve injury.

### 3.4 Baicalin prevented the up‐regulation of Trpv1 probably via ERK phosphorylation

**FIGURE 4:**
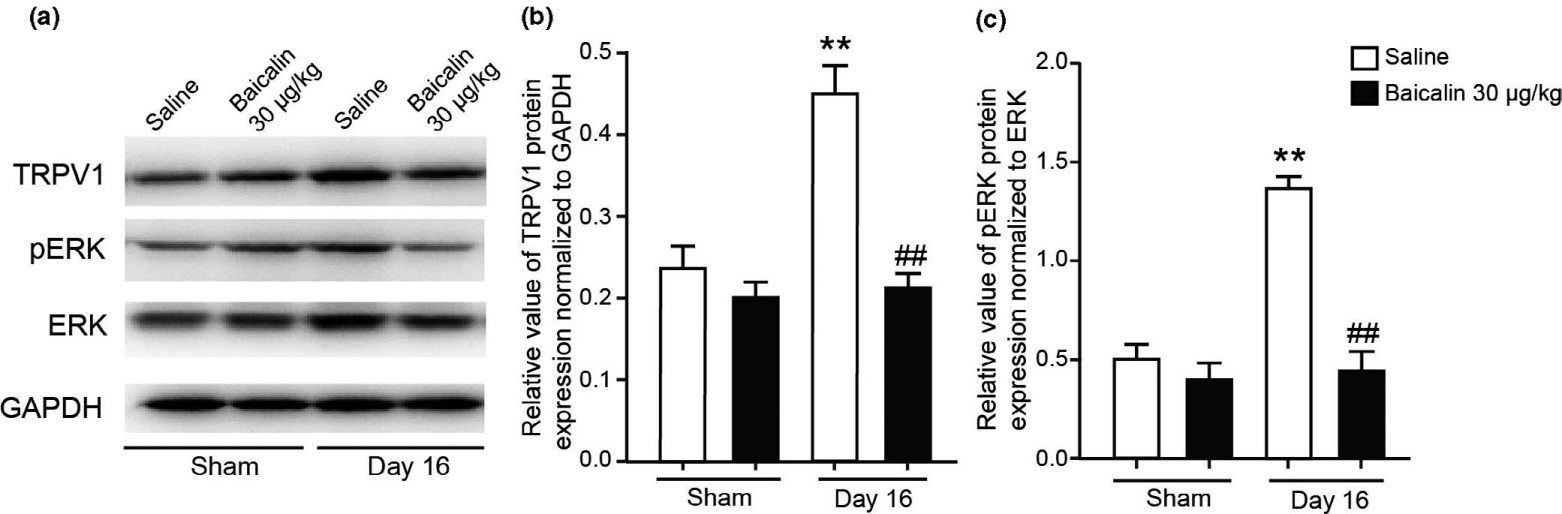
Protein expression of TRPV1 and ERK in DRG of CCI rats after consecutively baicalin treatment. (a) Representative images of western blot result of TRPV1, phosphorylation of ERK (pERK), ERK and GAPDH from DRG of CCI rats consecutively treated with baicalin (30 μg/kg) for 16 days. (b and c) Comparison of TRPV1 (b) and pERK (c) protein levels in DRG of CCI rats consecutively treated with baicalin (i.p.) for 16 days. Mean ± *SEM*, *n* = 8, ** *p* < .01 versus sham group, ^##^ *p* < .01 versus saline group. One‐way ANOVA followed by two‐tailed *t*‐test with Bonferroni correction

We then examined the expression of TRPV1 protein expression in DRG of rats. Western blot results demonstrated that the protein expression of TRPV1 was significantly increased in DRG of rats 16 days after CCI surgery, but this increase was prevented by repeated baicalin (30 μg/kg) administration in CCI rats (Figure 4a and b, *p* < .01, *n* = 8, one‐way ANOVA). We further asked the signalling pathway involved in the analgesic effects of baicalin. Western blot results demonstrated that the phosphorylation of ERK was significantly increased in DRG of CCI rats, but this increase did not happen in the DRG of CCI rats repeatedly treated with baicalin (30 μg/kg, Figure 4a and c, *p* < .01, *n* = 8, one‐way ANOVA). These results suggested that baicalin may relieve neuropathic pain by preventing TRPV1 up‐regulation and ERK phosphorylation in DRG of CCI rats.

## 4 DISCUSSION

Chronic neuropathic pain is characterized by rather well‐described symptoms of hyperalgesia (increased sensitivity to painful stimuli), allodynia (perception of innocuous stimuli as painful), altered sensation and spontaneous pain. Once peripheral nerve is damaged, a series of pathophysiologic events are induced including neuronal hyperexcitability, changes in perineuronal homeostasis, alternations in gene expression and a strong immune response (Sun et al., 2019). Meanwhile, subsequent events in central pain processing pathways consolidate and exaggerate the steady‐state pain condition (Kuner, 2010). Neuropathic pain is challenging to manage, while current treatment strategies often lack efficacy or have severe side‐effects in most patients. It has been reported that baicalin plays neuroprotective roles and relieves pain behavioural in animals with various types of nerve injury (Ates et al., 2006; Xu et al., 2018). The present study demonstrated that baicalin ameliorates neuropathic pain in a dose‐dependent manner. And baicalin suppressed TRPV1 up‐regulation upon peripheral nerve injury. In addition, baicalin reduced the phosphorylation of ERK in DRG of pSNL rats. Our results suggested the analgesic role of baicalin may be involved in a down‐regulation of TRPV1 and ERK phosphorylation in DRG of neuropathic pain rats.

Behavioural analysis revealed that repeated injection (i.p.) of baicalin alleviated either mechanical allodynia and thermal hyperalgesia in rats with peripheral nerve injury (Figure 2). TRP channels are reported to act as thermal, chemical and mechanical sensors (Julius, 2013). Accumulating evidences indicated that some TRP channels are responsible for neuropathic pain. TRPV1 is a well‐known channel that involved in nociception and thermosensation in peripheral and central nervous systems (Caterina et al., 1997). TRPV1 is shown to have a crucial role in nociceptive transmission under pathological forms of pain (Spicarova, Nerandzic, & Palecek, 2014). TRPV1 expression in nociceptors is altered in different models of neuropathy under pathological conditions (Biggs et al., 2007; Kim et al., 2008). In this study, we found that the mRNA expression of *Trpv1* and *Trpa1* expression level was dramatically increased in DRG after surgery, but only the up‐regulation of *Trpv1* was suppressed by baicalin administration (Figures 3 and 4), suggesting that baicalin may relieve neuropathic pain by modulating TRPV1 expression.

The mechanism of the analgesic role of baicalin in neuropathic pain has not yet been established. However, several papers may offer some evidences that could guide future investigation. Considerable evidences emphasized the importance of up‐regulation of TRPV1 in neuropathic pain (Spicarova et al., 2014). Our data revealed that the up‐regulation of TRPV1 expression level is significantly prevented by baicalin administration. Mitogen‐activated protein kinases (MAPK) are the main protein phospho‐regulating effectors that mediate nociceptive sensitization (Edelmayer, Brederson, Jarvis, & Bitner, 2014). It has been reported that TRPV1 is involved in endometriosis pain via ERK signalling pathway (Liu et al., 2018). Coincidently, our results demonstrated that baicalin prevents TRPV1 up‐regulation and ERK phosphorylation in DRG of CCI rats. TRPV1 may play an analgesic role in neuropathic pain signal transduction, which may be mediated by ERK signalling pathway. Moreover it has been reported that baicalin modulates the expression of PKC (Shi et al., 2018; Shou et al., 2017), which is the up‐stream of TRPV1 (Ozdemir, Naziroglu, Senol, & Ghazizadeh, 2016; Simões et al., 2016). Previous studies also demonstrated that baicalin reduces morphine tolerance by inhibiting microglial activation via AMPK signalling and down‐regulation of the expression of NF‐κB, IL1β, IL6 and tumour necrosis factor alpha (TNF‐α) (Han et al., 2014). In addition, pro‐inflammatory cytokines such as TNF‐α and IL‐1β activate signal transduction pathways in sensory neurons leading to downstream activation/sensitization of TRP channels (Chiu, Hehn, & Woolf, 2012). Baicalin has a strong anti‐inflammation effect (Cheng et al., 2017). Therefore, we could not exclude these possible mechanisms which baicalin may be involved.

In this study, administration of baicalin relieves mechanical and thermal pain without development of tolerance after 16 days of treatment, this may imply a potential feasibility for continuous treatment in clinical therapy. In conclusion, the present study established a novel role of baicalin as an analgesic agent for neuropathic pain. And the analgesic role of baicalin in neuropathic pain might involve a TRPV1‐ERK pathway. These results suggested that baicalin possesses analgesic efficacy against neuropathic pain and deserves further to confirm its effectiveness in clinically relevant painful diseases.

## CONFLICT OF INTEREST

The authors declare that they have no conflict of interest.

## AUTHOR CONTRIBUTION

**Zheyin Wang:** Data curation; Formal analysis; Methodology; Writing‐original draft. **Diyang Ling:** Conceptualization; Funding acquisition; Project administration; Writing‐review & editing. **Chengxiang Wu:** Data curation; Formal analysis. **Jian Han:** Data curation; Formal analysis; Methodology. **Yan Zhao:** Data curation; Formal analysis.

### Peer Review

The peer review history for this article is available at https://publons.com/publon/10.1002/VMS3.318.

## References

[vms3318-bib-0001] Ates, O. , Cayli, S. , Altinoz, E. , Gurses, I. , Yucel, N. , Kocak, A. , … Turkoz, Y. (2006). Effects of resveratrol and methylprednisolone on biochemical, neurobehavioral and histopathological recovery after experimental spinal cord injury. Acta Pharmacologica Sinica, 27, 1317–1325. 17007738 10.1111/j.1745-7254.2006.00416.x

[vms3318-bib-0002] Biggs, J. E. , Yates, J. M. , Loescher, A. R. , Clayton, N. M. , Boissonade, F. M. , & Robinson, P. P. (2007). Changes in vanilloid receptor 1 (TRPV1) expression following lingual nerve injury. European Journal of Pain, 11, 192–201. 16542859 10.1016/j.ejpain.2006.02.004

[vms3318-bib-0003] Caterina, M. J. , Schumacher, M. A. , Tominaga, M. , Rosen, T. A. , Levine, J. D. , & Julius, D. (1997). The capsaicin receptor: A heat‐activated ion channel in the pain pathway. Nature, 389, 816–824. 9349813 10.1038/39807

[vms3318-bib-0004] Chen, Y. , Willcockson, H. H. , & Valtschanoff, J. G. (2009). Influence of the vanilloid receptor TRPV1 on the activation of spinal cord glia in mouse models of pain. Experimental Neurology, 220, 383–390. 19815011 10.1016/j.expneurol.2009.09.030 PMC2784271

[vms3318-bib-0005] Cheng, P. , Wang, T. , Li, W. , Muhammad, I. , Wang, H. , Sun, X. , … Zhang, X. (2017). Baicalin alleviates lipopolysaccharide‐induced liver inflammation in chicken by suppressing TLR4‐mediated NF‐kappaB pathway. Frontiers in Pharmacology, 8, 547. 28868036 10.3389/fphar.2017.00547 PMC5563358

[vms3318-bib-0006] Cherng, C. H. , Lee, K. C. , Chien, C. C. , Chou, K. Y. , Cheng, Y. C. , Hsin, S. T. , … Wong, C. S. (2014). Baicalin ameliorates neuropathic pain by suppressing HDAC1 expression in the spinal cord of spinal nerve ligation rats. Journal of the Formosan Medical Association, 113, 513–520. 23684218 10.1016/j.jfma.2013.04.007

[vms3318-bib-0007] Chiu, I. M. , Hehn, C. A. V. , & Woolf, C. J. (2012). Neurogenic inflammation – The peripheral nervous system’s role in host defense and immunopathology. Nature Neuroscience, 15, 1063–1067. 22837035 10.1038/nn.3144 PMC3520068

[vms3318-bib-0008] Cho, J. H. , Jeong, M. Y. , Choi, I. S. , Lee, H. J. , & Jang, I. S. (2012). TRPA1‐like channels enhance glycinergic transmission in medullary dorsal horn neurons. Journal of Neurochemistry, 122, 691–701. 22671314 10.1111/j.1471-4159.2012.07817.x

[vms3318-bib-0009] Choi, S. I. , Lim, J. Y. , Yoo, S. , Kim, H. , & Hwang, S. W. (2016). Emerging role of spinal cord TRPV1 in pain exacerbation. Neural Plasticity, 2016, 5954890. 26885404 10.1155/2016/5954890 PMC4738952

[vms3318-bib-0010] Chou, T. C. , Chang, L. P. , Li, C. Y. , Wong, C. S. , & Yang, S. P. (2003). The antiinflammatory and analgesic effects of baicalin in carrageenan‐evoked thermal hyperalgesia. Anesthesia and Analgesia, 97, 1724–1729. 14633550 10.1213/01.ANE.0000087066.71572.3F

[vms3318-bib-0011] Edelmayer, R. M. , Brederson, J. D. , Jarvis, M. F. , & Bitner, R. S. (2014). Biochemical and pharmacological assessment of MAP‐kinase signaling along pain pathways in experimental rodent models: A potential tool for the discovery of novel antinociceptive therapeutics. Biochemical Pharmacology, 87, 390–398. 24300134 10.1016/j.bcp.2013.11.019

[vms3318-bib-0012] Finnerup, N. B. , Haroutounian, S. , Kamerman, P. , Baron, R. , Bennett, D. L. , Bouhassira, D. , … Jensen, T. S. (2016). Neuropathic pain: An updated grading system for research and clinical practice. Pain, 157, 1599–1606. 27115670 10.1097/j.pain.0000000000000492 PMC4949003

[vms3318-bib-0013] Han, Y. , Jiang, C. , Tang, J. , Wang, C. , Wu, P. , Zhang, G. , … Song, X. (2014). Resveratrol reduces morphine tolerance by inhibiting microglial activation via AMPK signalling. European Journal of Pain, 18, 1458–1470. 24756886 10.1002/ejp.511

[vms3318-bib-0014] Hao, Y. , Luo, X. , Ba, X. , Wang, J. , Zhou, S. , Yang, S. , … Sun, W. (2019). Huachansu suppresses TRPV1 up‐regulation and spinal astrocyte activation to prevent oxaliplatin‐induced peripheral neuropathic pain in rats. Gene, 680, 43–50. 30244138 10.1016/j.gene.2018.09.035

[vms3318-bib-0015] Julius, D. (2013). TRP channels and pain. Annual Review of Cell & Developmental Biology, 29, 355 10.1146/annurev-cellbio-101011-155833 24099085

[vms3318-bib-0016] Kim, H. Y. , Park, C. K. , Cho, I. H. , Jung, S. J. , Kim, J. S. , & Oh, S. B. (2008). Differential changes in TRPV1 expression after trigeminal sensory nerve injury. The Journal of Pain: Official Journal of the American Pain Society, 9, 280–288. 10.1016/j.jpain.2007.11.013 18226965

[vms3318-bib-0017] Kuner, R. (2010). Central mechanisms of pathological pain. Nature Medicine, 16, 1258–1266. 10.1038/nm.2231 20948531

[vms3318-bib-0018] Liu, Y. , Liu, W. , Wang, X. , Wan, Z. , Liu, Y. , & Leng, Y. (2018). Dexmedetomidine relieves acute inflammatory visceral pain in rats through the ERK pathway, toll‐like receptor signaling, and TRPV1 channel. Journal of Molecular Neuroscience, 66, 279–290. 30259406 10.1007/s12031-018-1172-5

[vms3318-bib-0019] Montell, C. , & Rubin, G. M. (1989). Molecular characterization of the Drosophila trp locus: A putative integral membrane protein required for phototransduction. Neuron, 2, 1313–1323. 2516726 10.1016/0896-6273(89)90069-x

[vms3318-bib-0020] Nilius, B. (2007). Transient receptor potential (TRP) cation channels: Rewarding unique proteins. Bulletin Et Memoires De L'academie Royale De Medecine De Belgique, 162, 244–253. 18075054

[vms3318-bib-0021] Ozdemir, U. S. , Naziroglu, M. , Senol, N. , & Ghazizadeh, V. (2016). Hypericum perforatum attenuates spinal cord injury‐induced oxidative stress and apoptosis in the dorsal root ganglion of rats: Involvement of TRPM2 and TRPV1 channels. Molecular Neurobiology, 53, 3540–3551. 26099309 10.1007/s12035-015-9292-1

[vms3318-bib-0022] Pohoczky, K. , Kun, J. , Szalontai, B. , Szoke, E. , Saghy, E. , Payrits, M. , … Helyes, Z. (2016). Estrogen‐dependent up‐regulation of TRPA1 and TRPV1 receptor proteins in the rat endometrium. Journal of Molecular Endocrinology, 56, 135–149. 26643912 10.1530/JME-15-0184

[vms3318-bib-0023] Shi, L. , Hao, Z. , Zhang, S. , Wei, M. , Lu, B. , Wang, Z. , & Ji, L. (2018). Baicalein and baicalin alleviate acetaminophen‐induced liver injury by activating Nrf2 antioxidative pathway: The involvement of ERK1/2 and PKC. Biochemical Pharmacology, 150, 9–23. 29338970 10.1016/j.bcp.2018.01.026

[vms3318-bib-0024] Shou, X. , Wang, B. , Zhou, R. , Wang, L. , Ren, A. , Xin, S. , & Zhu, L. (2017). Baicalin suppresses hypoxia‐reoxygenation‐induced arterial endothelial cell apoptosis via suppressing PKCdelta/p53 signaling. Medical Science Monitor : International Medical Journal of Experimental and Clinical Research, 23, 6057–6063. 29272263 10.12659/MSM.907989 PMC5747146

[vms3318-bib-0025] Simões, R. R. , dos Santos Coelho I. , do Espírito Santo C. C. , Morel, A. F. , Zanchet, E. M. , & Santos, A. R. (2016). Oral treatment with methanolic extract of the root bark of Condalia buxifolia Reissek alleviates acute pain and inflammation in mice: Potential interactions with PGE2, TRPV1/ASIC and PKA signaling pathways. Journal of Ethnopharmacology, 185, 319–326. 27013097 10.1016/j.jep.2016.03.050

[vms3318-bib-0026] Spicarova, D. , Nerandzic, V. , & Palecek, J. (2014). Update on the role of spinal cord TRPV1 receptors in pain modulation. Physiological Research, 63(Suppl 1), S225–S236. 24564662 10.33549/physiolres.932713

[vms3318-bib-0027] Sun, W. , Kou, D. , Yu, Z. , Yang, S. , Jiang, C. , Xiong, D. , … Hao, Y. (2019). A transcriptomic analysis of neuropathic pain in rat dorsal root ganglia following peripheral nerve injury. NeuroMolecular Medicine, 22(2), 250–263. 10.1007/s12017-019-08581-3 31858405

[vms3318-bib-0028] Sun, W. , Zhou, Q. , Ba, X. , Feng, X. , Hu, X. , Cheng, X. , … Jiang, C. (2018). Oxytocin relieves neuropathic pain through GABA release and presynaptic TRPV1 inhibition in spinal cord. Frontiers in Molecular Neuroscience, 11, 248. 30065629 10.3389/fnmol.2018.00248 PMC6056657

[vms3318-bib-0029] Sun, Y. Y. , Zhang, W. J. , Dong, C. L. , Zhang, X. F. , Ji, J. , Wang, X. , … Yuan, C. S. (2017). Baicalin alleviates nitroglycerin‐induced migraine in rats via the trigeminovascular system. Phytotherapy Research, 31, 899–905. 28488307 10.1002/ptr.5811

[vms3318-bib-0030] Xu, M. , Cheng, Z. , Ding, Z. , Wang, Y. , Guo, Q. , & Huang, C. (2018). Resveratrol enhances IL‐4 receptor‐mediated anti‐inflammatory effects in spinal cord and attenuates neuropathic pain following sciatic nerve injury. Molecular Pain, 14, 1744806918767549 29592782 10.1177/1744806918767549 PMC5881959

[vms3318-bib-0031] Xue, Q. , Jong, B. , Chen, T. , & Schumacher, M. A. (2007). Transcription of rat TRPV1 utilizes a dual promoter system that is positively regulated by nerve growth factor. Journal of Neurochemistry, 101, 212–222. 17217411 10.1111/j.1471-4159.2006.04363.x

[vms3318-bib-0032] Yuan, Q. L. , Guo, T. M. , Liu, L. , Sun, F. , & Zhang, Y. G. (2015). Traditional Chinese medicine for neck pain and low back pain: A systematic review and meta‐analysis. PLoS One, 10, e0117146 10.1371/journal.pone.0117146 25710765 PMC4339195

